# Growth hormone (GH) dose-dependent IGF-I response relates to pubertal height gain

**DOI:** 10.1186/s12902-015-0080-8

**Published:** 2015-12-18

**Authors:** Elena Lundberg, Berit Kriström, Bjorn Jonsson, Kerstin Albertsson-Wikland, on behalf of the study group

**Affiliations:** Institute of Clinical Science/Pediatrics, Umeå University, SE-90185 Umeå, Sweden; University of Uppsala, Women’s and Children’s Health, SE-75185 Uppsala, Sweden; Department of Physiology/Endocrinology, Institute of Neurosciences and Physiology, The Sahlgrenska Academy at University of Gothenburg, SE-40530 Gothenburg, Sweden

**Keywords:** Gain in height, IGF-I increment, IGF-I level, IGFBP3, Ratio IGF-I/IGFBP3, GH dose-dependent pubertal IGF-I response

## Abstract

**Background:**

Responsiveness to GH treatment can be estimated by both growth and ∆IGF-I. The primary aim of the present study was to investigate if mimicking the physiological increase during puberty in GH secretion, by using a higher GH dose could lead to pubertal IGFs in short children with low GH secretion. The secondary aim was to explore the relationship between IGF-I, IGFBP-3 and the IGF-I/IGFBP-3 ratio and gain in height.

**Methods:**

A multicentre, randomized, clinical trial (TRN88-177) in 104 children (90 boys), who had received GH 33 μg/kg/day during at least 1 prepubertal year. They were followed from GH start to adult height (mean, 7.5 years; range, 4.6–10.7). At onset of puberty, children were randomized into three groups, to receive 67 μg/kg/day (GH^67^) given once (GH^67x1^; *n* = 30) or divided into two daily injection (GH^33x2^; *n* = 36), or to remain on a single 33 μg/kg/day dose (GH^33x1^; *n* = 38). The outcome measures were change and obtained mean on-treatment IGF-I_SDS_, IGFBP3_SDS_ and IGF-I/IGFBP3 ratio_SDS_ during prepuberty and puberty. These variables were assessed in relation to prepubertal, pubertal and total gain in height_SDS_.

**Results:**

Mean prepubertal increases 1 year after GH start were: 2.1 IGF-I_SDS_, 0.6 IGFBP3_SDS_ and 1.5 IGF-I/IGFBP3ratio_SDS_. A significant positive correlation was found between prepubertal ∆IGFs and both prepubertal and total gain in height_SDS_. During puberty changes in IGFs were GH dose-dependent: mean pubertal level of IGF-I_SDS_ was higher in GH^67^ vs GH^33^ (*p* = 0.031). First year pubertal ∆IGF-I_SDS_ was significantly higher in the GH^67^vs GH^33^ group (0.5 vs −0.1, respectively, *p* = 0.007), as well as ∆IGF-I_SDS_ to the pubertal mean level (0.2 vs −0.2, *p* = 0.028). In multivariate analyses, the prepubertal increase in ‘∆IGF-I_SDS_ from GH start’ and the ‘GH dose-dependent pubertal ∆IGF-I_SDS_’ were the most important variables for explaining variation in prepubertal (21 %), pubertal (26 %) and total (28 %) gain in height_SDS_.

**Trial registration:**

TRN 88–177, not applicable 1988.

**Conclusion:**

The dose-dependent change in IGFs was related to a dose-dependent pubertal gain in height_SDS_. The attempt to mimic normal physiology by giving a higher GH dose during puberty was associated with both an increase in IGF-I and a dose-dependent gain in height_SDS_.

**Electronic supplementary material:**

The online version of this article (doi:10.1186/s12902-015-0080-8) contains supplementary material, which is available to authorized users.

## Background

Insulin-like growth factors (IGFs) have been used in the diagnosis of growth hormone (GH) deficiency, to monitor the impact of GH replacement therapy on growth and to assess treatment compliance and safety [1, 2]. Monitoring the impact of GH treatment on growth is mainly based on measurement of serum IGF-I levels, and less often on IGF-binding protein 3 (IGFBP3) levels and the IGF-I/IGFBP3 ratio. Rudman et al. were the first to report the relationship between short-term IGF-I increments and GH growth response [3]. Further short-duration studies in prepubertal children conducted by different groups found an increase in IGF-I from baseline to be a reliable sign of greater growth in response to GH [4–7]. Only two studies reported results for multiple variables (IGF-I, IGFBP3 and their molar ratio). These were non-randomized, 1-year clinical trials in prepubertal children with and without GH deficiency (GHD) [8, 9]. They both observed an increase in IGFs during GH treatment as a sign of high GH sensitivity and treatment compliance. To our knowledge, the relevance of the variables IGF-I, IGFBP3 and the IGF-I/IGFBP3 ratio in relation to prepubertal, pubertal and total gain in height has not been previously reported in GH-treated children.

Under normal conditions, serum IGF-I level increases slowly during childhood before rising to a peak in puberty. This peak correlates with pubertal stage due to the action of sex steroids to increase GH secretion [10–13]. Factors explaining pubertal growth in response to GH therapy: gender, age, the difference between the child’s height standard deviation score (SDS) and midparental height_SDS_ (diffH-MPH) at the onset of puberty, and GH dose were identified from the KIGS observational study, but the IGF-I variable was not available in that study [14]. There have only been two published randomized trials in GH-deficient pubertal children on GH treatment receiving different GH doses. Both reported a greater pubertal height gain in high GH-dose groups (50–100 μg/kg/d) accompanied by an increase in IGF-I [15, 16]. Our group has recently published results from a randomized study in non-GH-deficient children followed from early puberty to adult height (AH): data show the greater the increase in IGF-I, the greater the gain in height [17]. In children with low GH secretion diagnosed with idiopathic isolated GHD (IIGHD), we have also reported that GH dosing, mimicking the physiological pubertal increase in GH secretion, has a dose-dependent effect on the gain in height_SDS_ until AH. In this randomized GH-treatment trial, pubertal height gain_SDS_ and AH_SDS_ were greater in children randomized to a high GH dose (67 μg/kg/d) than a standard dose (33 μg/kg/d) [18, 19]. The hypothesis of the present analysis of prospectively collected data in the aforementioned clinical trial was that the IGFs would follow the normal pubertal change in GH-deficient children receiving a higher, more physiological, GH dose during puberty. The secondary aim was to explore the relationship between serum IGF-I, IGFBP-3 and the IGF-I/IGFBP-3 ratio and gain in height until AH.

## Patients and methods

### Ethics

The study (TRN number 88–177) was approved by the Ethics Committees at the Universities of Gothenburg, Lund, Linköping, Uppsala, Huddinge and Umeå and by the Karolinska Institute (dnr LU 426–1988). Informed consent was obtained from the children and their parents verbally with written registration date in the patients’ medical file.

### Study design

The study was a nationwide, randomized, multicenter trial conducted from 1988 to 2009 and its design has been described previously [18, 19]. In brief, all children with IIGHD had received GH at a dose of 33 μg/kg for at least 1 year prior to the onset of puberty. After the development of clinical signs of puberty, the children were allocated randomly, without stratification, into three dose groups: 33 μg/kg once daily (GH^33x1^); 67 μg/kg once daily (GH^67x1^) or 33 μg/kg twice daily (GH^33x2^). The children were followed until AH (the observed height at a growth rate of < 1 cm during the preceding 12 months). They were seen at least once a year at a university hospital to monitor treatment safety and efficacy and at their local children’s hospital at 3-monthly intervals.

### GH dose reduction

In line with the protocol, it was accepted that any patient could reduce GH dose or stop treatment if he/she was satisfied with the height development. The reduced doses used were 25 % less than the randomized dose (doses were 50 μg/kg/d and 25 μg/kg/d for the high and low dose groups, respectively). In the intention-to-treat (ITT) population the GH dose was reduced in 35 children and GH treatment was stopped in 5 children; this affected 44 % of children in the high dose and 29 % in the standard dose groups. For efficacy analyses, only IGF-I_SDS_ levels obtained during treatment with the randomized dose were used, but for safety analyses all IGF-I_SDS_ measurements were used.

### Patients

#### Inclusion and exclusion criteria

All patients were diagnosed with IIGHD in the 1980s. GH deficiency was diagnosed based on a GH response cut off corresponding to “10 μg/L” [20, 21] in two GH provocation tests, mainly the arginine–insulin tolerance test (AITT). A positive response to GH treatment was also an inclusion criterion. This was ascertained by an increase in height velocity of at least 50 % during the first prepubertal year of GH treatment (33 μg/kg/d). IGF-I, IGFBP3 and the IGF-I/IGFBP3 ratio_SDS_ were measured but were not required for diagnosis. Children with any hormone insufficiency in addition to GH or with significant chronic diseases or syndromes were excluded from the study.

#### Safety population

The study population has been described previously [19]. Briefly: a total of 149 (116 boys) short children (< −2SDS) with low GH_max_ during AITT (GH_max_AITT) were enrolled in the study between 1989 and 2000 and form the safety population. Of the enrolled children, 38 were excluded from efficacy analyses due to protocol violation/wrong inclusion: 1 child had a bone age (BA) delay of 3.6 years at GH start, 24 children were already pubertal (breast > 1, testes > 8 ml) at or within 1 year after GH start and 1 child was lost from follow-up. Moreover, 6 children who were born at a gestational age < 32 weeks and 6 adopted children with missing information at birth were excluded from the present analyses. Seven further children (6 boys) for whom most IGF-I data were missing were also excluded from the present analysis.

#### ITT/PP population

The remaining 104 children (90 boys) constituted the ITT population of whom 95 (82 boys) comprised the per-protocol (PP) population.

Of the 9 children (8 boys) not belonging to the PP population, 5 boys had stopped GH treatment prematurely (<2.25 years after study start and before AH was reached) and 4 children (3 boys) on GH^67^ decreased their GH dose to GH^33x1^

When the study group was divided into the groups to which they were later randomized, there were no differences in IGF levels between the groups. Moreover, there were no differences in IGF-I levels at baseline between boys and girls.

## Methods

### Hormone measurements

*Serum IGF-I and IGFBP3* levels were measured using an IGFBP-blocked radioimmuno assay (RIA) with an excess of IGF-II for determination of IGF-I, and a specific RIA for IGFBP3 (Mediagnost GmbH, Tübingen, Germany). The intra-assay coefficients of variation (CVs) for the IGF-I assay were 11.1, 7.2 and 7.4 % at concentrations of 36, 204 and 545 μg/L, respectively; the interassay CVs for the same concentrations were 13.5, 8.8, and 9.9 %, respectively. For the IGFBP3 assay, the intra-assay CVs were 7.1, 7.3, and 7.9 % at concentrations of 1800, 3790 and 5776 μg/L, respectively; the interassay CVs for the same concentrations were 13.4, 10.5 and 14.1 %, respectively. Results were converted into SDS according to age, sex and pubertal stage, and the ratio_SDS_ of IGF-I to IGFBP3 was calculated [12, 22]. IGF-I and IGFBP3 were analyzed before and after the initiation of GH treatment (+10 days, +1 month, +3 months, +1 year and annually thereafter). For the purpose of the present analyses, mean pubertal IGF-I_SDS_, IGFBP3_SDS_ and IGF-I/IGFBP3 ratio_SDS_ were calculated based on individual mean levels in the time period from 12 months after study start to treatment stop. The change in level was defined as the mean level (as calculated above) minus the level at study start. The prepubertal mean IGF-I_SDS_, IGFBP3_SDS_, and IGF-I/IGFBP3 ratio_SDS_ were based on data collected in the time period from 1 year after GH start until the clinical onset of puberty. All samples from each individual were analyzed at the same time.

#### Growth hormone

GH_max_ was assessed using both the AITT and a spontaneous 24-h GH secretion profile [20, 21]. GH concentrations were analysed with polyclonal antibodies, and for comparison, all measurements were converted to the WHO standard international reference preparation 80/505, even if measured with 66/127 [20].

#### Growth outcome

The outcomes used for evaluation of growth response were: prepubertal, pubertal and total gain in height_SDS_ calculated as previously described [19, 23].

*Pubertal gain in height*_*SDS*_ was defined as AH_SDS_ minus last recorded pre-pubertal height_SDS,_ the SDS estimated with the childhood component of the total reference [23, 24].

*Prepubertal gain in height*_*SDS*_ was defined as height_SDS_ at last pre-pubertal visit minus height_SDS_ at GH start using the childhood component of the total reference [24].

*Total gain in height*_*SDS*_ was calculated using AH_SDS_ (adult height in cm transferred into SDS for age 18 years) minus height_SDS_ at GH start, using the prepubertal childhood component of the total growth reference [23, 24].

There were no differences in gain in height_SDS_ between the two high-dose groups with GH^67^ given once or divided into twice daily GH^33^ (Table 1); therefore, the results from the high-dose groups are presented combined (GH^67+33x2^).

**Table 1:** Growth characteristics at adult height according to randomization group

At Adult Height	33 × 1	67 × 1	33 × 2	67 × 1 + 33 × 2
	*n* = 38	*n* = 30	*n* = 36	*n* = 66
ITT all, *n* = 104	Mean ± SD	Mean ± SD	Mean ± SD	Mean ± SD
Age, years	18.6 ± 1.5	18.6 ± 1.27	18.6 ± 1.79	18.6 ± 1.56
Adult height_SDS_	−1.2 ± 0.68	−0.9 ± 0.93	−0.8 ± 0.88	−0.8 ± 0.89
diffH-MPH_SDS_	0.2 ± 0.80	0.3 ± 1.13	0.2 ± 1.00	0.2 ± 1.05
Total gain in height_SDS_	1.6 ± 0.83	2.1 ± 1.14^*^	1.9 ± 0.82	2.0 ± 0.97^*^
Prepubertal gain in height_SDS_	1.2 ± 0.85	1.4 ± 1.28	1.2 ± 0.71	1.3 ± 1.00
Pubertal gain in height_SDS_	0.4 ± 0.58	0.7 ± 0.90	0.7 ± 0.77	0.7 ± 0.83^*^
Years from puberty	5.7 ± 1.22	5.5 ± 1.51	5.9 ± 1.58	5.7 ± 1.55
Years from GH start	8.5 ± 2.87	8.7 ± 3.21	8.9 ± 2.92	8.8 ± 3.03
Years on GH	7.6 ± 2.88	7.7 ± 2.99	7.7 ± 3.04	7.7 ± 2.99
Years in study	5.1 ± 1.24	4.9 ± 1.48	5.1 ± 1.54	5.0 ± 1.50
PP, boys, *n* = 82	*n* = 34	*n* = 23	*n* = 25	*n* = 48
Age, years	18.7 ± 1.42	18.8 ± 1.14	18.8 ± 1.89	18.8 ± 1.56
Adult height_SDS_	−1.2 ± 0.60	−0.8 ± 0.84	−0.8 ± 0.93	−0.8 ± 0.88
diffH-MPH_SDS_	0.2 ± 0.82	0.4 ± 1.05	0.2 ± 1.00	0.3 ± 1.02
Total gain in height_SDS_	1.5 ± 0.69	2.0 ± 0.88^*^	1.9 ± 0.75a	1.9 ± 0.81^**^
Prepubertal gain in height_SDS_	1.00 ± 0.61	1.1 ± 0.58	1.2 ± 0.74	1.1 ± 0.67
Pubertal gain in height_SDS_	0.4 ± 0.60	0.9 ± 0.88^*^	0.7 ± 0.75	0.8 ± 0.81^*^
Years from puberty	5.7 ± 1.18	5.6 ± 1.55	6.2 ± 1.81	5.9 ± 1.7
Years from GH start	8.3 ± 2.62	8.5 ± 2.98	9.1 ± 3.25	8.8 ± 3.11
Years on GH	7.5 ± 2.62	7.5 ± 2.78	8.0 ± 3.16	7.8 ± 2.96
Years in study	5.1 ± 1.16	4.9 ± 1.45	5.4 ± 1.73	5.1 ± 1.6
ITT, Girls, *n* = 14	*n* = 3	*n* = 5	*n* = 6	*n* = 11
Age, years	16.7 ± 1.46	17.2 ± 1.14	17.3 ± 1.31	17.2 ± 1.18
Adult height_SDS_	0.0 ± 0.49	−0.6 ± 0.94	−1.3 ± 0.71^*^	−1.0 ± 0.87
diffH-MPH_SDS_	0.2 ± 0.50	0.3 ± 0.95	−0.2 ± 0.96	0.0 ± 0.94
Total gain in height_SDS_	3.0±0.98	3.1±1.80	1.6±0.97	2.2±1.54
Prepubertal gain in height_SDS_	2.9±1.50	3.1±2.29	1.1±0.51	2.0±1.83
Pubertal gain in height_SDS_	0.3±0.33	−0.1±0.71	0.4±0.89	0.2±0.82
Years from puberty	4.5±0.54	5.8±1.55	5.6±0.91	5.7±1.18
Years from GH start	10.8±5.46	10.9±3.90	8.1±1.72	9.4±3.12
Years on GH	10.1±4.92	9.4±3.95	7.1±1.50	8.1±2.97
Years in study	4.1±1.24	5.5±1.58	4.6±1.09	5.0±1.34

*SDS* standard deviation score, *MPH* midparental height, *diffH-MPH*_*SDS*_ the difference between the childs height_SDS_ vs MPH_SDS_

^*^ *p* = 0.001; ^**^ *p* = 0.05

### Normal or delayed infancy–childhood transition (ICT)

Age at ICT was available for 92 of the 104 children. A delayed ICT (DICT; ICT at > 12 months of age) was found in 33 children (11 boys) [25] (Additional file 1: Table S2).

#### Familial short stature

Midparental height (MPH) was below −2 SDS [26] in 23 children (21 boys).

#### Small for gestational age

Twenty children (18 boys) were born small for gestational age with a birth length_SDS_ and/or birth weight_SDS_ < −2 SDS [27].

### Statistical analyses

Statistical analyses were performed using the standard package SPSS version 20. Results are expressed as mean ± SD unless otherwise specified. Analyses concerning primary and secondary outcome variables were performed using non-parametric tests of the Wilcoxon type (Wilcoxon signed-rank test for within-group and Mann–Whitney *U* test for between-group comparisons). Safety analyses included all 149 children who received study drug. Analyses were performed for the ITT population and for boys in the PP population. Statistical significance was considered if *p* < 0.05.

Simple bivariate correlation analyses were performed using Pearson’s r.

*Stepwise multiple regression analysis* was used to analyse the influence of IGF-I variables on height gain and AH_SDS_. Data on birth characteristics and growth until 3 years of age, as well as baseline characteristics and prepubertal growth, were added as predictors in the analysis. Only variables entering the regressions below the significance level *p* < 0.05 were used. No correction was performed for multiplicity. Stepwise forward regression analyses were performed with *p* < 0.05 as entering criterion for predictors and *p* < 0.10 for exclusion after inclusion in an earlier step.

## Results

### Patient characteristics

The characteristics of the study group have recently been reported including 7 more children [19] (see Table 1, Additional file 1: Table S1 for characteristics according to later randomization groups). Girls were younger than boys at GH start, 7.4 vs 10.2 years (*p* = 0.002) and also at study start, 12.3 vs 13.7 years (*p* = 0.001). Girls gained more height_SDS_ during prepuberty than boys, 2.2 vs 1.1 SDS, respectively (*p* = 0.0069) and less during puberty 0.2 vs 0.7, respectively (*p* = 0.029) (Table 1; Additional file 1: Table S2).

#### Comparison between children with DICT and normal ICT

Patients with DICT had significantly lower IGF-I_SDS_ at GH start than patients with a normal ICT, −1.9 ± 2.0 vs −0.9 ± 0.9 (*p* = 0.014). See Additional file 1: Table S2 for baseline and study characteristics according to gender and ICT.

#### Per-protocol population (PP)

Comparing ITT and PP populations, similar results were found for the boys in both groups (data not shown); therefore, only the ITT population will be presented (Table 1).

### Prepubertal study results

#### IGF-I_SDS_ (Table 2) IGFBP3_SDS_ (Table 3) and IGF-I/IGFBP3 ratio_SDS_ (Table 4)

**Table 2:** IGF-I during GH treatment according to randomization group

ITT	All		33 × 1		67 × 1		33 × 2		67 × 1 + 33 × 2
IGF-I_SDS_	n	Mean ± SD	n	Mean ± SD	n	Mean ± SD	n	Mean ± SD	Mean ± SD
At GH start	71	−1.2 ± 1.59	32	−1.4 ± 1.51	20	−0.9 ± 1.66	19	−1.1 ± 1.66	−1 ± 1.64
1^st^ year after GH start	76	0.9 ± 1.71	28	0.9 ± 1.37	24	1 ± 1.95	24	0 ± 1.89	0.9 ± 1.9
ΔIGF-I_SDS_, 1^st^ year after GH start	63	2.1 ± 1.48	25	2.2 ± 1.37	19	1.9 ± 1.94	19	2.1 ± 1.1	2 ± 1.56
Last pre-pubertal	90	1.2 ± 1.23	35	1.1 ± 1.15	28	1.1 ± 1.33	27	1.4 ± 1.26	1.3 ± 1.29
IGF-I_SDS_ prepubertal level	100	0.8 ± 1.07	37	0.6 ± 1.08	30	0.9 ± 1.05	33	1 ± 1.08	1 ± 1.06
Study start	104	0.9 ± 1.21	38	1 ± 1.02	30	0.8 ± 1.33	36	1 ± 1.29	0.9 ± 1.31
1^st^ year after study start	100	1.2 ± 1.07	36	0.9 ± 0.88	30	1.3 ± 1.15	34	1.5 ± 1.13^*^	1.4 ± 1.13^**^
ΔIGF-I_SDS_ 1^st^ year after study start	100	0.3 ± 0.98	36	−0.1 ± 0.96	30	0.5 ± 1.09^****^	34	0.4 ± 0.78^****^	0.5 ± 0.93^*^
IGF-I_SDS_ pubertal level	104	1 ± 0.95	38	0.9 ± 0.67	30	1.1 ± 1.14	36	1.1 ± 1.03	1.1 ± 1.07
ΔIGF.I_SDS_Pubertal level from study start	104	0.1 ± 0.92	38	−0.2 ± 0.86	30	0.4 ± 1.1^***^	36	0.1 ± 0.75^*****^	0.2 ± 0.92
Before study stop	104	0.6 ± 1.36	38	0.6 ± 1.18	30	0.9 ± 1.29	36	0.3 ± 1.57	0.6 ± 1.46
After study stop	65	−0.4 ± 1.54	23	−0.1 ± 0.91	18	−0.3 ± 1.4	24	−0.6 ± 2.04	−0.5 ± 1.78

*Δ* change, *IGF-I* insulin-like growth factor I, *SDS* standard deviation score

^*^ *p* = 0.007; ^**^ *p* = 0.011; ^***^ *p* = 0.014; ^****^ *p* = 0.021; ^*****^ *p* = 0.028

**Table 3:** IGFBP3SDS during GH treatment according to randomization group

ITT	All		33 × 1		67 × 1		33 × 2		67 × 1 + 33 × 2
IGFBP3_SDS_	n	Mean ± SD	n	Mean ± SD	n	Mean ± SD	n	Mean ± SD	Mean ± SD
At GH start	71	−0.3 ± 0.76	32	−0.4 ± 0.8	20	−0.2 ± 0.66	19	−0.3 ± 0.83	−0.3 ± 0.74
1^st^ year after GH start	76	0.3 ± 0.82	28	0.3 ± 0.73	24	0.4 ± 0.87	24	0.1 ± 0.87	0.2 ± 0.87
ΔIGFBP3_SDS_ 1^st^ year after GH start	63	0.6 ± 0.55	25	0.7 ± 0.47	19	0.7 ± 0.57	19	0.5 ± 0.64	0.6 ± 0.6
Prepubertal level	100	0.3 ± 0.54	37	0.3 ± 0.54	30	0.3 ± 0.63	33	0.3 ± 0.63	0.3 ± 0.54
Last pre-pubertal	90	0.4 ± 0.55	35	0.5 ± 0.49	28	0.4 ± 0.48	27	0.4 ± 0.68	0.4 ± 0.58
Study start	104	0.4 ± 0.63	38	0.4 ± 0.58	30	0.3 ± 0.63	36	0.4 ± 0.67	0.3 ± 0.65
1^st^ year after study start	100	0.3 ± 0.56	36	0.3 ± 0.43	30	0.3 ± 0.58	34	0.4 ± 0.67	0.4 ± 0.63
ΔIGFBP3_SDS_1^st^ year after study start	100	0 ± 0.45	36	−0.1 ± 0.49	30	0 ± 0.51	34	0 ± 0.36	0 ± 0.43
Pubertal level	104	0.3 ± 0.56	38	0.2 ± 0.45	30	0.3 ± 0.62	36	0.3 ± 0.63	0.3 ± 0.62
ΔIGFBP3_SDS_ Pubertal level from study start	104	−0.1 ± 0.43	38	−0.2 ± 0.45^**^	30	0.0 ± 0.51	36	−0.1 ± 0.32	−01 ± 0.42^*^
Before study stop	104	0.2 ± 0.77	38	0.2 ± 0.82	30	0.3 ± 0075	36	0.1 ± 0.75	0.2 ± 0.75
After study stop	64	0.4 ± 0.8	23	0.6 ± 0.57	17	0.3 ± 0ö65	24	0.2 ± 1.01	0.2 ± 0.87

*Δ* change, *IGFBP3* IGF-binding protein 3, *SDS* standard deviation score

*p = 0.029; **p = 0.038

**Table 4:** IGF-I/IGFBP3 RatioSDS during GH treatment according to randomization group

ITT	All		33 × 1		67 × 1		33 × 2		67 × 1 + 33 × 2
IGF-I/IGFBP3 RatioSDS	n	Mean ± SD	N	Mean ± SD	n	Mean ± SD	n	Mean ± SD	Mean ± SD
At GH start	71	−1 ± 1.08	32	−1.1 ± 0.94	20	−0.8 ± 1.24	19	−0.8 ± 1.13	−0.8 ± 1.17
1^st^ year after GH start	76	0.6 ± 1.2	28	0.5 ± 1.21	24	0.6 ± 1.31	24	0.7 ± 1.13	0.6 ± 1.21
ΔRatio_SDS_ 1^st^ year after GH start	63	1.5 ± 1.24	25	1.6 ± 1.25	19	1.2 ± 1.6	19	1.6 ± 0.74	1.4 ± 1.24
Prepubertal level	100	0.4 ± 0.95	37	0.2 ± 0.98	30	0.5 ± 0.97	33	0.6 ± 0.89	0.6 ± 0.92
Last pre-pubertal	90	0.7 ± 1.18	35	0.5 ± 1.11	28	0.6 ± 1.24	27	1 ± 1.17	0.8 ± 1.22
Study start	104	0.5 ± 0.98	38	0.6 ± 0.99	30	0.3 ± 1.06	36	0.6 ± 0.89	0.5 ± 0.97
1^st^ year after study start	100	0.9 ± 0.87	36	0.6 ± 0.87	30	0.9 ± 0.86	34	1.2 ± 0.78^*^	1.1 ± 0.83^**^
ΔRatio_SDS_ 1^st^ year after study start	100	0.4 ± 0.93	36	0 ± 0.95	30	0.6 ± 1.03	34	0.6 ± 0.73^****^	0.6 ± 0.87^***^
Pubertal level	104	0.9 ± 0.78	38	0.7 ± 0.74	30	0.9 ± 0.82	33	1 ± 0.78	0.9 ± 0.79
ΔRatio_SDS_ Pubertal level from study start	104	0.3 ± 0.89	38	0.1 ± 0.92	30	0.6 ± 1.04	36	0.3 ± 0.66	0.5 ± 0.86
Before study stop	104	0.5 ± 1.1	38	0.5 ± 0.88	30	0.7 ± 1.14	36	0.3 ± 1.26	0.5 ± 1.21
After study stop	64	−0.7 ± 1.03	23	−0.9 ± 0.73	17	−0.7 ± 1.18	24	−0.5 ± 1.16	−0.6 ± 1.16

*Δ* change, *IGF-I* insulin-like growth factor I, *IGFBP3* IGF-binding protein 3, *SDS* standard deviation score

*p = 0.003; **p = 0.008; ***p = 0.015; ****p = 0.020

At GH start, mean IGF-I_SDS_ was −1.2 for the total study group, mean IGFBP3_SDS_ was −0.3 and mean IGF-I/IGFBP3 ratio_SDS_ was–1.0, and values did not differ between the three groups who later constituted the randomization groups (Additional file 1: Table S2).

The observed prepubertal mean change (∆) in IGF-I_SDS_ from GH start to the first year on GH therapy was 2.1 (Table 2). The corresponding ∆IGFBP3_SDS_ was 0.6 (Table 3) and the ∆IGF/IGFBP-3 ratio_SDS_ was 1.5 (Table 4). The change in IGF-I_SDS_ relative to prepubertal mean level was 2.21, range −3.89 to 6.55.

#### The relationship between prepubertal IGF variables and gain in height_SDS_ for all children on GH^33^ (Tables 5, 6 and 7)

**Table 5:** IGF-I variables and their correlations with different gain in height outcomes

ITT		Pubertal gain in height_SDS_		Total gain in height_SDS_		Prepubertal gain in height_SDS_	
IGF-I_SDS_	n	r	p	r	p	r	p
At GH start	71	0.02		−0.37	0.002	−0.53	0.001
1^st^ year after GH start	76	0.11		−0.14		−0.30	0.009
AIGF-I_SDS_1^st^ year after GH start	63	0.25	0.053	0.42	0.001	0.29	0.019
Last pre-pubertal	90	−0.01		−0.01		0.00	
IGF-I_SDS_ prepubertal level	100	−0.09		−0.10		−0.02	
Study start	104	−0.11		−0.11		−0.01	
1^st^ year after study start	100	0.14		−0.03			
AIGF-I_SDS_ 1^st^ year after study start	100	0.26	0.010	0.10			
Pubertal level	104	0.21	0.034	−0.08			
AIGF-I_SDS_Pubertal level from study start	104	0.35	0.001	0.05			
Before study stop	104	0.26	0.008	−0.17	0.083		
After study stop	65	0.37	0.002	−0.24	0.059		

*Δ* change, *IGF-I* insulin-like growth factor I, *SDS* standard deviation score

**Table 6:** IGFBP3 variables and their correlations with gain in height outcomes

ITT		Pubertal gain in height_SDS_		Total gain in height_SDS_		Prepubertal gain in height_SDS_	
IGFBP3_SDS_	n	r	p	r	p	r	p
At GH start	71	−0.05		−0.36	0.002	−0.43	0.001
1^st^ year after GH start	76	0.09		−0.22	0.052	−0.39	0.001
∆IGFBP3_SDS_1^st^ year after GH start	63	0.13		0.29	0.022	0.24	0.057
Last pre-pubertal	90	−0.14		−0.05		0.07	
IGFBP3_SDS_Prepubertal level	100	−0.12		−0.11		0.00	
Study start	104	−0.09		−0.03		0.06	
1^st^ year after study start	100	0.09		−0.02			
∆IGFBP3_SDS_1^st^ year after study start	100	0.20	0.044	0.04			
Pubertal level	104	0.13		0.01			
∆IGFBP3_SDS_Pubertal level from study start	104	0.30	0.002	0.05			
Before study stop	104	0.32	0.001	−0.09			
After study stop	64	0.35	0.005	−0.17			

*Δ* change, *IGFBP3* IGF-binding protein 3, *SDS* standard deviation score

**Table 7:** IGF-I/IGFBP3 ratioSDS variables and their correlations with gain in height outcomes

ITT		Pubertal gain in height_SDS_		Total gain in height_SDS_		Prepubertal gain in height_SDS_	
IGF-I/IGFBP3 Ratio _SDS_	n	r	p	r	p	r	p
At GH start	71	−0,16		−0,37	0,001	−0,31	0,009
Ratio_SDS_1^st^ year after GH start	76	0,09		0,01		−0,08	
∆Ratio_SDS_1^st^ year after GH start	63	0,28	0,026	0,42	0,001	0,24	0,061
Ratio_SDS_ Prepubertal level	100	0,03		−0,09		−0,10	
Last pre-pubertal	90	0,08		0,01		−0,06	
Study start	104	−0,02		−0,19	0,048	−0,16	0,095
1^st^ year after study start	100	0,16		−0,11			
∆Ratio_SDS_1^st^ year after study start	100	0,16		0,08			
Ratio_SDS_ Pubertal level	104	0,23	0,021	−0,19	0,048		
∆Ratio_SDS_ Pubertal level after study start	104	0,22	0,022	0,04			
Before study stop	104	0,07		−0,14			
After study stop	64	0,15		0,05			

*Δ* change IGF-I/IGFBP3 Ratio, *IGF-I* insulin-like growth factor I, *IGFBP3* IGF-binding protein 3, *SDS* standard deviation score

The group means for the three IGF variables at GH start were negatively correlated with prepubertal gain in height_SDS_ (IGF-I_SDS_*r* = −0.53, *p* = 0.001; IGFBP-3_SDS_*r* = −0.43, *p* = 0.001; IGF-I/IGFBP3 ratio_SDS_*r* = −0.31, *p* = 0.009) and with total gain in height_SDS_ (IGF-I_SDS_*r* = −0.37, *p* = 0.002; IGFBP3_SDS_*r* = −0.36, *p* = 0.002; IGF-I/IGFBP-3 ratio_SDS_*r* = −0.37, *p* = 0.001).

**Fig. 1:**
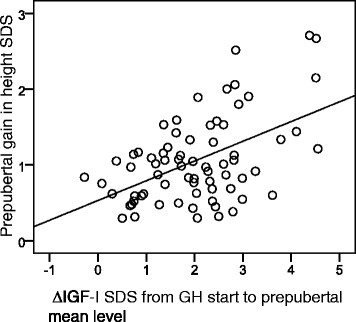
Prepubertal change in IGF-I_SDS_ in relation to prepubertal gain in height_SDS_. The change (∆) in IGF-I_SDS_ from GH start to prepubertal mean level in relation to prepubertal gain in height_SDS_ (*r* = 0.26, *p* < 0.001)

The first-year prepubertal ∆IGF-I_SDS_ was positively correlated with the prepubertal gain in height_SDS_ (Fig. 1), but did not correlate with the ∆IGFBP3_SDS_ or ∆IGF-I/IGFBP3 ratio_SDS_ after 1 year. All three prepubertal first-year ∆IGF variables were positively correlated with the total gain in height_SDS_ (∆IGF-I_SDS_*r* = 0.42, *p* = 0.001; ∆IGFBP3_SDS_*r* = 0.29, *p* = 0.022 and ∆IGF-I/IGFBP3 ratio_SDS_*r* = 0.42, *p* = 0.001).

### Pubertal study results

#### Pubertal IGF-I_SDS_, IGFBP3_SDS_ and IGF-I/IGFBP3 ratio_SDS_ mean study levels (Tables 2, 3 and 4)

At study start, the group mean IGF-I_SDS_ was 0.9, the group mean IGFBP3_SDS_ was 0.4 and the group mean IGF-I/IGFBP3 ratio_SDS_ was 0.5. There were no differences between the three randomization groups.

The highest IGF-I_SDS_ was reached after 1 year in the study and the value subsequently decreased, i.e. the pubertal mean level was lower than the value at 1 year after randomization (see Table 2).

The pubertal IGF-I_SDS_ for the total study group ranged from −2.1 to 3.5, with mean value being higher for the GH^67^group than for the GH^33^group, 1.1 vs 0.9, respectively (*p* = 0.031). The mean IGFBP3_SDS_ for the total study group was 0.3, with a range −1.2 to 1.3. For IGF-I/IGFBP3 ratio_SDS_, the mean was 0.9, with a range of −1.3 to 2.4. There were no significant differences between the randomization groups for IGFBP3_SDS_ or IGF-I/IGFBP3 ratio_SDS_.

#### Dose-dependent change (∆) in IGF-I_SDS_ (Table 2), IGFBP3_SDS_ (Table 3) and IGF-I/IGFBP3 ratio_SDS_ (Table 4)

After the first year in the study, pubertal ∆IGF-I_SDS_ was significantly greater in the group randomized to GH^67^ than in the group still receiving the GH^33^ dose, 0.5 vs −0.1, respectively (*p* = 0.007; Table 2).

A similar pattern was evident for the first year ∆IGF-I/IGFBP3 ratio_SDS_ for GH^67^ vs GH^33^, 0.6 vs 0, respectively (*p* = 0.015; Table 4), but there was no change in IGFBP3_SDS_ after 1 year for any group (Table 3).

**Fig. 2:**
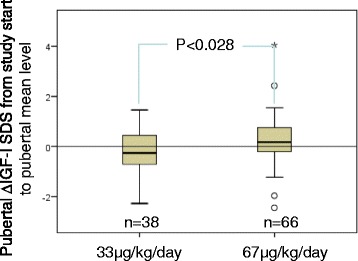
Pubertal change in IGF-I_SDS_ according to randomization dose. Pubertal change (∆) in IGF-I_SDS_ from study start to pubertal mean level according to GH dose, 33 μg/kg/day vs 67 μg/kg/day. Box and whisker plots showing median, interquartile range (IQR) and values within ±1.5 IQR are given

When instead calculating the ∆SDS from study start to the mean pubertal level, again *∆*IGF-I_SDS_ was significantly greater for the GH^67^ vs GH^33^ group, 0.2 vs −0.2, respectively (*p* = 0.028), as shown in Fig. 2. When comparing the mean prepubertal and pubertal level of IGF-I_SDS_, IGFBP3_SDS_ and IGF-I/IGFBP3 ratio_SDS_ actually 47, 65 and 34 % respectively of children, equally for both dose groups, did not maintain the prepubertal level during puberty.

#### The relationship between IGF-I_SDS_, IGFBP3_SDS,_ IGF-I/IGFBP3 ratio_SDS_ and gain in height_SDS_, (Tables 5, 6 and 7)

At randomization, the mean IGF-I_SDS_ and IGFBP3_SDS_ did not correlate with any of the height gain outcomes, whereas the mean IGF-I/IGFBP3 ratio_SDS_ was negatively associated with total height gain_SDS_ (*r* = −0.19, *p* = 0.048; Tables 5, 6 and 7).

**Fig. 3:**
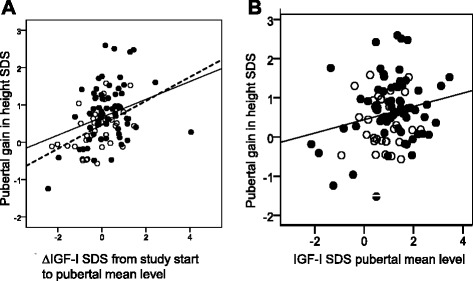
Pubertal change in IGF-I_SDS_ left and IGF-I_SDS_ pubertal mean level right according to pubertal gain in height_SDS_. a Change in pubertal IGF-I_SDS_ from study start to pubertal mean level in relation to pubertal gain in height_SDS_. Open circles GH^33^, *r* = 0.32, *p* < 0.003; dots GH^67^. Correlation for total group: *r* = 0.24, *p* < 0.026, with no significant slope for either GH^33^ or GH^67^ separately. b Attained IGF-I_SDS_ pubertal mean level in relation to pubertal gain in height_SDS_. Open circles GH^33^; dots GH^67^. Correlation for total group: *r* = 0.17, *p* < 0.034, with no significant slope for either GH^33^ or GH^67^ separately

For the individual, the pubertal ∆IGF_SDS_ from randomization was significantly correlated with the pubertal gain in height_SDS_ for both GH^33^ and GH^67^, *r* = 0.32 (*p* = 0.003) and *r* = 0.24 (*p* = 0.026) respectively (Fig. 3a). For the total study group, the IGF-I_SDS_ pubertal mean level correlated with the pubertal gain in height_SDS_, *r* = 0.17 (*p* = 0.034; Fig. 3b). The range in gain in height_SDS_ was wide, and there were no significant differences between the dose groups; therefore, the results from the total study group were used for further analyses.

Disregarding randomization dose, the pubertal gain in height_SDS_ was positively correlated with the 1st year pubertal ∆IGF-I_SDS_ (*r* = 0.26, *p* = 0.010) and also with the ∆IGF-I_SDS_ to the pubertal mean level (*r* = 0.35, *p* = 0.001, Table 5).

The first year pubertal ∆IGFBP3_SDS_ correlated with the pubertal gain in height_SDS_ (*r* = 0.20, *p* = 0.044), and the mean pubertal ∆IGFBP3_SDS_ correlated with the pubertal gain in height level (*r* = 0.30, *p* = 0.002; Table 6). The attained mean pubertal level of IGFBP3_SDS_ was not correlated with height gain outcomes. The pubertal ∆IGF-I/IGFBP3 ratio_SDS_ was correlated (*r* = 0.22, *p* = 0.022) with the pubertal gain in height_SDS_. The attained mean pubertal level of IGF-I/IGFBP3 ratio_SDS_ correlated with the pubertal gain in height_SDS (_*r* = 0.23, *p* = 0.021) and with total gain in height_SDS_ (*r* = −0.19, *p* = 0.048; Table 7).

### Multivariate regression (Tables 8 and 9)

**Table 8:** Multivariate analyses on height outcomes with GH 67 μg/kg/day and IGF variables available

ITT	B	SEB	p	R^2^
Total gain in height_SDS_				
(Constant)	1.23	0.148	0.000	28
IGF-I_SDS_ 1 year after GH start	0.24	0.058	0.000	
∆IGF-I_SDS_ pubertal level from study start	0.29	0.093	0.003	
Pubertal gain in height_SDS_				
(Constant)	0.42	0.121	0.001	26
∆IGF-I_SDS_ pubertal level from study start	0.34	0.086	0.000	
∆IGF-I/IGFBP3 ratio_SDS_ 1 year after GH start	0.20	0.063	0.002	
Prepubertal gain in height_SDS_				
(Constant)	0.91	0.136	0.000	21
IGF-I_SDS_ at GH start	−0.28	0.069	0.000	

*Δ* change, *IGF-I* insulin-like growth factor I, *IGFBP3* IGF binding protein 3, *SDS* standard deviation score

**Table 9:** Multivariate analyses on height outcomes with all variables available

ITT	B	seb	P	R^2^, %
Total gain in height_SDS_				
(Constant)	0.19	0.200	0.340	63
Prepubertal gain in height_SDS_	0.55	0.085	0.000	
Bone age delay at GH start	−0.25	0.072	0.001	
∆IGF-I/IGFBP3 ratio_SDS_ 1 year after GH start	0.20	0.065	0.003	
High dose	0.38	0.161	0.023	
Pubertal gain in height_SDS_				
(Constant)	0.31	0.183	0.095	46
Bone age delay study start	−0.26	0.069	0.000	
Years on GH prepubertal	−0.11	0.029	0.001	
∆IGF-I_SDS_ 1 year after GH start	0.14	0.049	0.008	
∆IGF-I_SDS_ pubertal level from study start	0.19	0.083	0.025	
Prepubertal gain in height_SDS_				
(Constant)	−0.63	0.089	0.000	94
Years on GH, prepubertal	0.28	0.013	0.000	
∆Height_SDS_ 1 year after GH start	0.69	0.074	0.000	
∆IGF-I/IGFBP3ratio_SDS_ 1 year after GH start	0.12	0.029	0.000	
Gender (female)	0.53	0.100	0.000	
Bone age delay at GH start	−0.10	0.029	0.002	
∆IGFBP3_SDS_ 1 year after GH start	0.14	0.062	0.030	

*A* change, *IGF-I* insulin-like growth factor I, *IGFBP3* IGF-binding protein 3, *Ratio* IGF-I/IGFBP3 Ratio, *SDS* standard deviation score

#### Variance in pubertal ∆IGFs

In total, 39 % of the variation in pubertal ∆IGF-I_SDS_ was explained by the variable ‘IGF-I_SDS_ at study start’; the lower the value at study start, the greater the increase during puberty. Similarly, for pubertal ∆IGFBP3_SDS_, 28 % (also including age, +) was explained and for ∆IGF-I/IGFBP3 ratio_SDS_ 40 % was explained.

#### Variance in gain in height_SDS_ with only IGF variables available (Table 8)

With only the IGF variables available, 26 % of the variation in *pubertal* gain in height_SDS_ could be explained by ‘pubertal (from study start to pubertal level) ∆IGF-I_SDS_’ and ‘first year prepubertal ∆IGF-I/IGFBP3 ratio_SDS_’. For *total* gain in height_SDS_, 28 % of the variation was explained by ‘prepubertal IGF-I_SDS_ first year after GH start’ and ‘pubertal ∆IGF-I_SDS_’. Regarding the variation in *prepubertal* gain in height_SDS,_ 21 % was explained by ‘IGF-I_SDS_ at GH start’ (the lower the better).

#### Variance in gain in height_SDS_ with all variables available (Table 9)

The variables are presented in the order they entered in the analyses. For the *pubertal gain* in height_SDS_, 46 % of the variation could be explained by ‘bone age at study start’ (−), ‘prepubertal years on GH’ (−), ‘∆IGF-I_SDS_ 1 year after GH start’ (+) and ‘pubertal ∆IGF-I_SDS_ (pubertal level from study start).

For the variation in *total gain* in height_SDS_. 63 % could be explained by the variables ‘prepubertal gain in height_SDS_’ (+), ‘bone age at GH start’ (−),’ ∆IGF/IGFBP3 ratio_SDS_ 1 year after GH start’ (+) and ‘high GH dose’ (+).

In the analysis of *prepubertal gain* in height_SDS_, 94 % of the variation was explained by ‘number of prepubertal years on GH’, ‘first year ∆height_SDS_’, ‘first year ∆IGF-I/IGFBP3 ratio_SDS_’_, ‘_gender’ (girl +), ‘bone age at GH start’ (the greater the delay the better) and ‘first year ∆IGFBP3_SDS_’.

## Discussion

### Changes in IGFs relate to prepubertal and pubertal height gain

The present analysis reported results for multiple variables including IGF-I, IGFBP3 and their ratio in IIGHD children from the start of GH treatment until AH, and the relationship of IGFs to both the prepubertal period when all participants received the same 33 μg/kg/d and the pubertal period when they were randomised to 33 or 67 μg/kg/d. The analysis focused on GH responsiveness as estimated by changes and obtained prepubertal and pubertal levels of IGF-I_SDS_, IGFBP3_SDS_ and IGF-I/IGFBP3 ratio_SDS_. The main findings were: a significant dose-dependent (33 or 67 μg/kg/d) change in IGF-I_SDS_ from randomization at onset of puberty to mean pubertal study level, and a positive correlation between this pubertal ∆IGF-I_SDS_ and the pubertal gain in height_SDS_. The greatest change was found in IGF-I followed by a less pronounced change in IGF-I/IGFBP3 ratio, while IGFBP3 values remained more stable. The GH dose given during puberty did not maintain the mean prepubertal IGF-I level, suggesting that some children may have benefitted from a higher dose in order to undergo a pubertal growth spurt of normal magnitude [19]. The need for wide-ranging individual GH dosing in order to promote growth has previously been demonstrated during prepuberty [28], here we find similar requirements when studying IGFs and growth during the pubertal period. Actually, GH responsiveness in the present study group was so broad that the low dose was too high for some individuals while the high dose was too low for others in order to attain IGFs and pubertal height gain within normal range.

Prepubertal responsiveness to GH, reflected by a significant increase in IGFs after GH start [5, 28], has previously been reported to be of great importance for the short-term growth response; in the present analysis it was found to be important also for the total gain in height. This highlights the importance of individual GH dosing from treatment start, with the available prediction models presently being the best tools with which to estimate outcomes [29, 30]. Thus, when an increased GH dose during puberty induced a greater ∆IGF-I_SDS_, this resulted in a greater pubertal height gain than observed with a standard GH dose for most subjects. This finding has previously been reported in non-GHD subjects, with ∆IGF-I_SDS_ found to be the most reliable variable correlating with pubertal gain in height_SDS_ [17]. Thus, there is no principal difference in the IGF-I response and growth response between these two aetiologies of short stature, although the magnitude of GH responsiveness is higher in the GH-deficient than in the non-GH-deficient group [5, 31].

GH has effects on longitudinal bone growth both directly at the growth plate and locally mediated through IGF-I [32, 33]. The increase in IGF-I level as a response to GH treatment could be seen as a sign of GH responsiveness [5]. GH/IGF-I responsiveness varies not only between individuals but also between tissues within an individual, e.g. more GH is needed to produce an effect on IGF-I production than for longitudinal bone growth [34].

### Pubertal response in IGFs

In the present analyses, there was a significant difference in pubertal IGF-I_SDS_ between randomization groups, with higher mean values in the GH^67^ than the GH^33^ group. This finding supports results from the study of Mauras et al. [15] who reported that by using higher GH doses during puberty (100 vs 42 μg/kg/d), a significantly higher pubertal gain in height_SDS_ was found, as well as a higher but non-significant IGF-I response in the high- relative to the low-dose group. The difference in importance of IGF-I in relation to our study may be due to differences in IGF-I references: we used our in-house reference with SDS considering gender, age and pubertal stage [12]. In the study by Sas et al. using GH doses corresponding to 25 or 50 μg/kg/d until AH, the mean increase in IGF-I_SDS_ after 1 year on GH was twice as high for the high- versus the low-dose group, although the difference did not reach significance. The relationship between IGF-I_SDS_ and growth response was not given [16]. Neither IGFBP3 nor IGF-I/IGFBP3 ratio was measured in these two published trials.

#### The multivariate analyses

In agreement with our findings in non-GH-deficient children [17], among the IGF-variables also in the present study, the pubertal ∆IGF-I_SDS_ was found to be the most informative variable, and more important than the level per se, for explaining the variance in both pubertal and total gain in height_SDS_ . When all variables were allowed, bone age delay and less prepubertal years on GH in addition to ∆IGF-I_SDS_ was positive for *pubertal gain in height*_*SDS*_. This may be explained by some remaining catch-up growth occurring during puberty in some study subjects, even though all children had been treated with GH for at least 1 year before randomization.

The explanatory variables for pubertal gain in height were in accordance with those identified within the KIGS observational study, except that bone age was selected in our study and chronological age in the KIGS study [14]. This difference may be because bone age was estimated by a single radiologist in our trial which increased the quality and consistency of this variable.

For *total gain in height* in the present study, a GH dose high enough to result in a substantial prepubertal ∆IGF-I/IGFBP-3 ratio_SDS_ was favourable. The high dose was set at 67 μg/kg/d even though the present results suggest that nearly 50 % of the children could have benefitted from an even higher dose. We need to remember that at the time of study design, there were limited data on GH treatment doses and safety, and no tools available for estimation of individual GH responsiveness such as the prediction models for GH growth response [30, 35].

### Prepubertal response

Mean IGF-I_SDS_, IGFBP3_SDS_ and IGF-I/IGFBP3 ratio_SDS_ at GH start were in the low/normal range. Lower IGF levels at baseline were associated with greater changes after 1 year of GH therapy. We found a strong negative bivariate linear correlation between low baseline IGF-I_SDS_, IGFBP3_SDS_, their ratio_SDS_ and both prepubertal and total gain in height_SDS_. The prepubertal IGF-I response and its relationship to gain in height is already well known [3–5, 36]. It was previously reported that the observed prepubertal 1-year growth response makes it possible to estimate the full prepubertal gain in height in both children with GHD and in non-GH-deficient groups [37]. Knowledge of the relationship between the change in IGFs and total gain in height_SDS_ can now be added, and highlights the importance of considering prepubertal GH responsiveness and achieving a greater first year ∆IGFs when selecting GH dose. The ∆IGF-I_SDS_, ∆IGFBP3_SDS_ and ∆IGF/IGFBP3 ratio_SDS_ 1 year after GH start mirrored individual GH responsiveness as measured by IGF-I generation. This supports results from 1-year non-randomized observations in GH-deficient and non-GH-deficient groups [8, 9, 36].

In our analyses, prepubertal IGF-I_SDS_ did not correlate with gain in height. This is in contrast to the findings of the study by Cohen et al. who found a relationship between IGF-I level (the higher the better) and height gain in prepubertal children treated for 2 years with IGF-I-targeting GH doses [28]. The difference in results may be explained by differences in study design, mainly in terms of inclusion criteria and dose ranges. However, in both studies the ∆IGF-I_SDS_ was found in multivariate analyses to be the most informative variable explaining growth response.

#### The multivariate analyses

In multivariate regression analyses using only IGF variables, baseline IGF-I_SDS_ alone explained 21 % of the variation in prepubertal gain in height. When adding auxological variables, the variation explained improved to 94 % with the following important variables: bone age delay at GH start (more delay better growth) and prepubertal years on GH (more years, more growth). Many prepubertal years on GH are also a sign of GHD (being young at diagnosis was associated with more severe GHD).

### Different information from IGFBP3_SDS_ and IGF/IGFBP3 ratio_SDS_

IGFBP3 and IGF-I/IGFBP3 ratio are not routinely monitored during GH treatment. IGFBP3 is less sensitive to short-term nutritional variations and diseases than IGF-I and could therefore be valuable when monitoring efficacy of and compliance with GH treatment [38]. In addition, a more pronounced change in IGF-I relative to IGFBP3 results in an increased IGF-I/IGFBP3 ratio [22, 39], which can be seen as an indicator of increased IGF-I bioavailability [40]. In the present study, changes in IGF-I/IGFBP3 ratio_SDS_ followed changes in IGF-I, and correlated significantly with both pubertal and total gain in height_SDS_. In the multivariate regression analysis, prepubertal ∆IGF-I/IGFBP3 ratio_SDS_ was selected for explanation of variance in both prepubertal and total gain in height_SDS_. ∆IGF-I/IGFBP3 ratio_SDS_ may be a result of a synergistic effect of IGF-I and IGFBP3 during GH therapy [41], where IGFBP3 modulates the actions of IGF-I, as well as having an independent effect [40]. To our knowledge, longitudinal observation of IGF-I/IGFBP3 ratio_SDS_ during pubertal growth in a population with IIGHD has not previously been reported.

### IGFs markers for safety

In the present study no dose-dependent adverse event involving carbohydrate metabolism was observed, which confirms previous studies with high GH doses [15, 16, 42, 43]. However, a modest association between increased circulating level of IGF-I and an increased risk of common cancers in adult has been reported [1, 44]. Most circulating IGF-I is bound to IGFBP3 and to the acid labile subunit (ALS) [45], therefore, the IGF-I/IGFBP3 ratio_SDS_ could reflect the tissue availability of IGF-I and its correlation with free IGF-I [39, 40, 46]. Our data showed no dose-dependent differences in pubertal IGF-I/IGFBP3 ratio_SDS_, only a broad range of IGF-I_SDS_ and IGFBP3_SDS_, highlighting that GH dosing needs to be individualized [31].

### Compliance

In the present study, stable IGF-I_SDS_ and IGFBP3_SDS_ concentrations were observed, which is a sign of good compliance [38, 47]. There were more than 10 samples for each patient and nearly 2000 samples in the total analysis. Only 8 single samples were excluded due to suspected poor compliance.

Poor compliance could be a factor that underestimates the study results; both in terms of IGF-I and growth responses. Pubertal teenagers are known to have the lowest treatment compliance due to many factors, not least psychological [48, 49]. In order to promote good compliance in our study, all participants were responsible for their own injections and were followed every third month. At each visit they were invited to discuss their treatment with their endocrine team [50].

### The heterogeneity of the study group

The study group was heterogeneous, including patients with classic GHD, partial GHD and some short boys with low GH secretion in the late prepubertal period [51], and there was a broad range of IGF-I_SDS_ and IGFBP3_SDS_ at baseline. This reflects the reality in daily clinical practice and allows results to represent patients with a wider range of baseline levels of GH secretion and a broad range in GH responsiveness.

### Limitations of the study

The study was designed before individual GH responsiveness was broadly considered. Children were randomized to weight-based dosing and 38 % of patients decided to reduce or stop GH treatment before AH was reached due to satisfaction with their attained height, which was accepted by the protocol. However, the combination of the limited number of patients in the treatment groups, the broad variability in their growth responses, and the premature stop/reduction of GH dose will underestimate the result of the study regarding changes in IGF-I_SDS_, IGFBP-3_SDS_ and IGF-I/IGFBP-3 ratio_SDS_, as well as the dose-dependent effect on pubertal gain in height_SDS_.

The onset of puberty was defined by clinical signs in the present trial, making it possible that some pubertal growth had occurred before randomization, thus leading to underestimation of the pubertal IGF-I response and gain in height.

Girls constituted a small group in the current analyses. The low number of girls in each dose group does not allow conclusions about use of an even higher GH dose during puberty in girls.

## Conclusion

In the present analysis we studied GH responsiveness estimated on the obtained levels and change of IGF-I_SDS_, IGFBP3_SDS_ and IGF-I/IGFBP-3 ratio_SDS_ and the associated growth response in children with IIGHD randomized in puberty to different weight-based GH dose regimens. Thereby, the relationship of IGFs to gain in height_SDS_ during both the prepubertal and pubertal growth phases could be explored. The prepubertal increase in IGFs associated with a 33 μg/kg/d GH dose and the GH dose-dependent (33 or 67 μg/kg/d) pubertal increase in IGF-I, were both important variables that explained the total gain in height: the higher the prepubertal GH responsiveness, the greater the total gain in height_SDS_. Our hypothesis that increased GH dose during puberty would result in a more pronounced IGF-I response and greater growth was found to be valid: the higher GH dose during puberty was followed by both higher IGF-I_SDS_ and a greater gain in height_SDS_ than observed in patients receiving the lower GH dose. Thus, of great importance in the clinical setting: the individual who remains short at onset of puberty require a GH dose increase great enough to result in an increment in IGF-I in order to gain any height_SDS_ during puberty.

## Additional file

Additional file 1:
**Table S1.** Baseline characteristics according to randomization group: at birth, at GH start and at study start. **Table S2.** Baseline characteristics according to gender and infancy–childhood transition (ICT): at birth, at GH start, at study start and at adult height. (PDF 222 kb)

## Abbreviations

AITT: Arginine–insulin tolerance test
AH: Adult height
BL: Birth length
BMI: Body mass index
BW: Body weight
BA: Bone age
DICT: Delayed infancy–childhood transition, ICT >12 months after birth
diffH-MPH_SDS_: The difference between the child’s Height_SDS_ vs MPH_SDS_
GH: Growth hormone
GHD: Growth hormone deficiency
GH^33x1^: GH, Standard dose 33 μg/kg once daily injection
GH^33x2^: GH, 33 μg/kg twice daily injection
GH^67x1^: GH, 67 μg/kg once daily injection
GH^67^: GH 33×2 and 67×1, the high-dose group
GH_max_24h: Maximum GH level during a spontaneous 24h GH profile
GH_max_AITT: Maximum GH level during an AITT
ICT: Infancy–childhood transition, months
IGF-I: Insulin-like growth factor I
IIGHD: Idiopathic isolated growth hormone deficiency
IGFBP-3: Insulin-like growth factor-binding protein 3
IRP: International reference preparation
ITT: Intention-to-treat
MPH: Midparental height
PP: Per-protocol
RIA: Radioimmuno assay
SDS: Standard deviation score
WHO: World Health Organization
