# Supplementary material for: Growth hormone (GH) dose-dependent IGF-I response relates to pubertal height gain
Source: BMC Endocr Disord. 2015 Dec 18;15:84. doi: 10.1186/s12902-015-0080-8 (PMC4683753; doi:10.1186/s12902-015-0080-8)
Supplement: Additional file 1: — Table S1. Baseline characteristics according to randomization group: at birth, at GH start and at study start. Table S2. Baseline characteristics according to gender and infancy–childhood transition (ICT): at birth, at GH start, at study start and at adult height. (PDF 222 kb) [file 12902_2015_80_MOESM1_ESM.pdf]

Supplementary Table 1. Baseline characteristics according to randomization group: at birth, at GH start and at study start.

	33x1	67x1	33x2	67x1+33x2
**IIGHD, n=104**	**n=38**	**n=30**	**n=36**	**n=66**
**ITT, all**	**Mean±SD**	**Mean±SD**	**Mean±SD**	**Mean±SD**
At birth				
BL_{SDS}	-0.9±0.95	-1.1±1.24	-0.7±1.25	-0.9±1.5
BW_{SDS}	-0.8±1.14	-0.8±1.35	-0.5±1.41	-0.7±1.8
Gestational age, weeks	39.1±2.16	39.4±1.52	39.4±1.73	39.4±1.3
Midparental $height_{SDS}$	-1.3±1.00	-1.2±0.97	-1±0.93	-1.1±0.4
Mother's $height_{SDS}$	-1.0±0.82	-0.6±1.15	-1±0.94	-0.8±1.4
Father's $height_{SDS}$	-1.2±1.09	-1.2±0.88	-0.7±0.86[ab]	-0.9±0.9
$\Delta Height_{SDS}$ 0-3 years	-1.3±0.96	-1.3±1.23	-1.7±1.31	-1.5±1.8
At GH start				
Age at GH start, years	10.1±3.1	9.9±3.54	9.6±3.04	9.8±3.5
Bone age delay, years	-1.7±1.10	-1.8±0.86	-1.9±1.3	-1.8±1.
$\Delta Height_{SDS}$, pretreatment year	-0.2±0.69	-0.2±0.66	-0.1±0.25	-0.1±0.8
$Height_{SDS}$	-2.7±0.54	-3±0.83	-2.8±0.63	-2.9±0.3
BMI_{SDS}	-0.6±0.87	-0.6±1.07	-0.5±1.1	-0.6±1.7
diffH-MPH_{SDS}	-1.3±1.06	-1.7±1.17	-1.7±0.85[a]	-1.7±1[d]
IGF-I_{SDS}	-1.4±1.51	-1.2±1.25	-1.1±1.66	-1.1±1.5
GHmaxAITT, mU/L	18.0±9.03	17.1±11.1	18.6±10.92	17.9±10.3
GHmax24h, mU/L	32.1±19.55	26.6±16.8	37.4±24.62	32.3±21.8
GHmaxAITT/24h, mU/L	20.6±15.04	17.9±12.13	22.5±16.18	20.4±14.6
1 year after GH start				
$Height_{SDS}$ 1st treatment year	-0.2±0.57	-2.1±0.64	-2±0.66	-2±0.5
ΔH_{SDS}, 1st treatment year	0.7±0.26	0.9±0.79	0.8±0.28	0.8±0.7
At study start (Randomization)				
Age, years	13.5±1.32	13.7±1.56	13.4±1.24	13.5±1.39
Bone age delay, years	-1.1±0.93	-1.2±1.00	-1.3±1.32	-1.3±1.7
IGF-I_{SDS}	1.0±1.02	0.8±1.33	1±1.29	0.9±1.31
Years from puberty onset	0.6±0.50	0.7±1,12	0.8±0.78	0.7±0.94
ΔHeight, to randomisation, cm	6±4.03	7.1±8,14	7.8±5.93	7.5±6.97
ΔBone age from GH start, years	0.6±1.09	0.5±0,86	0.6±1	0.5±0.93

[a]p=0.034; [b]p=0.022; [c]p=0.028; [d]p=0.021

BW, birth weight; BL, birth length; BMI, body mass index; GH_{max}AITT/24h, maximum GH level during AITT (arginin–insulin tolerance test) or during a spontaneous 24h GH profile; ΔHeight, gain in height; IGF-I, insulin-like growth factor I; MPH, midparental height; diffH-MPH_{SDS}, the difference between the child's $height_{SDS}$ vs MPH_{SDS}; SDS, standard deviation score.

Supplementary Table 2. Baseline characteristics according to gender and infancy–childhood transition (ICT): at birth, at GH start, at study start and at adult height.

ITT	Boys n=90	Girls n=14	ALL n=104		Normal ICT n=59	DICT n=33	
	**Mean±SD**	**Mean±SD**	**Mean±SD**	**p**	**Mean±SD**	**Mean±SD**	**p**
At birth							
BL_{SDS}.	-0.9±1.12	-0.7±1.29	-0.9±1.14	0.871	-1.1±1.15	-0.6±0.89	0.086
BW_{SDS}.	-0.8±1.3	-0.2±1.16	-0.7±1.29	0.209	-1±1.26	-0.3±1.14	0.014
Gestational age, weeks	39.2±1.91	39.6±1.28	39.3±1.84	0.541	39.2±1.79	39.3±1.9	0.648
Midparental $height_{SDS}$	-1.2±0.96	-0.8±0.98	-1.2±0.97	0.199	-1.2±0.92	-1.1±1.02	0.974
Mother's $heigh_{tSDS}$	-0.9±0.92	-0.4±1.18	-0.9±0.97	0.177	-0.9±1	-0.8±0.94	0.616
Father's $height_{SDS}$	-1±0.97	-0.9±1.05	-1±0.98	0.710	-1±0.87	-1±1.12	0.899
$\Delta Height_{SDS}$ 0-3 years	-1.3±1.05	-2±1.73	-1.4±1.17	0.072	-1.2±1.25	-1.9±0.93	0.001
At GH start							
Age at GH start, years	10.2±3	7.4±3.37	9.9±3.19	0.002	10.3±3	8.5±3.28	0.006
Bone age delay, years	-1.8±0.99	-1.6±1.76	-1.8±1.1	0.766	-1.8±1.18	-1.7±1.02	0.392
$\Delta Height_{SDS}$, pretreatment year	-0.1±0.27	-0.5±1.37	-0.1±0.57	0.732	-0.2±0.7	-0.1±0.33	0.555
$Height_{SDS}$	-2.7±0.55	-3.2±1.13	-2.8±0.67	0.103	-2.7±0.69	-3±0.58	0.006
BMI_{SDS}	-0.6±0.99	-0.4±1.08	-0.6±1	0.331	-0.8±0.93	-0.3±1.09	0.027
$diffH\text{-}MPH_{SDS}$	-1.5±1	-2.1±1.16	-1.6±1.04	0.081	-1.4±1	-1.8±1.07	0.075
$IGF\text{-}I_{SDS}$	-1.2±1.5	-1.5±1.25	-1.3±1.47	0.577	-0.9±0.95	-1.9±2.04	0.014
GHmaxAITT, mU/L	18.6±10.38	14±8.7	17.9±10.23	0.180	19.8±10.22	14.8±11.22	0.030
GHmax24h, mU/L	31.1±19.63	41.3±27.41	32.2±20.57	0.409	34.9±19.2	30.8±23.64	0.371
GHmaxAITT/24h, mU/L	21±14.96	17.5±12.67	20.5±14.66	0.403	21.8±13.6	19.7±18.34	0.130
1 year after GH start					.		
$Height_{SDS}$ 1st treatment year	-2±0.58	-2.2±0.83	-2±0.62	0.782	-2±0.55	-2.2±0.65	0.051
$\Delta Height_{SDS}$, 1st treatment year	0.7±0.27	1±1.13	0.8±0.48	0.481	0.7±0.58	0.8±0.29	0.368
At study start (Randomization)							
Age, years	13.7±1.25	12.3±1.45	13.5±1.36	0.001*	13.5±1.36	13.4±1.42	0.754
Bone age delay, years	-1.3±1.09	-0.9±1.07	-1.2±1.09	0.209	-1.3±1.1	-1.1±1.11	0.281
$IGF\text{-}I_{SDS}$	1±1.12	0.5±1.63	0.9±1.21	0.371	0.9±1.32	0.8±1.12	0.386
Years from puberty onset	0.7±0.84	0.6±0.62	0.7±0.81	0.826	0.6±0.48	0.9±1.07	0.246
ΔHeight to randomization cm	7±6.34	6.4±4.21	7±6.08	0.917	6±4.01	8.6±7.59	0.092
ΔBone age from GH start, years	0.5±1	0.8±0.92	0.6±0.99	0.534	0.5±1.06	0.7±0.81	0.474
ΔHeight vs GH start, cm	24.6±15.64	38.2±26.22	26.3±17.75	0.048	23.1±16.57	34.5±19.07	0.002
At Adult Height							
Age, years	18.8±1.46	17.1±1.2	18.6±1.53	0.000	18.5±1.28	18.4±1.65	0.906
Adult $height_{SDS}$	-1±0.82	-0.8±0.9	-1±0.83	0.469	-1±0.68	-0.9±1	0.515
$diffH\text{-}MPH_{SDS}$	0.2±0.98	0±0.85	0.2±0.96	0.376	0.2±0.89	0.2±1.16	0.594
Total gain in $height_{SDS}$	1.8±0,82	2.4±1.45	1.8±0.94	0.105	1.7±0.92	2.1±0.96	0.048
Prepubertal gain in $height_{SDS}$	1.1±0.65	2.2±1.74	1.2±0.95	0.006	1.1±1.02	1.5±0.91	0.022
Pubertal gain in $height_{SDS}$	0.7±0.75	0.2±0.73	0.6±0.76	0.029	0.6±0.71	0.6±0.83	0.760
Years from puberty onset	5.8±1.47	5.4±1.17	5.7±1.3	0.545	5.6±1.29	5.9±1.35	0.299

Years from GH start	8.5±2.86	9.7±3.53	8.7±2.96	0.261	8.2±2.7	10±3.26	0.008
Years on GH at AH	7.5±2.86	8.6±3.35	7.6±2.94	0.170	7.1±2.52	9±3.45	0.008
Years in study after rand	5.1±1.42	4.8±1.33	5±1.41	0.523	5±1.34	5.1±1.4	0.874

*Sign by definition
BW, birth weight; BL, birth length; BMI, body mass index; DICT, delayed infancy–childhood transition; GH_{max}AITT/24h, maximum GH level during AITT (arginin-insulin tolerance test) or during a spontaneous 24h GH profile; ΔHeight, gain in height; IGF-I, insulin-like growth factor I; MPH, midparental height; diffH-MPH_{SDS}, the difference between the childs $height_{SDS}$ vs MPH_{SDS}; SDS, standard deviation score.
